# 1235. Anti-HCV Prevalence and Associated Risk Factors in Patients of Psychiatry Hospitals in Latvia

**DOI:** 10.1093/ofid/ofac492.1067

**Published:** 2022-12-15

**Authors:** Ieva Tolmane, Inga Upmace, Inga Azina, Baiba Rozentale

**Affiliations:** Riga East University Hospital, Rīga, Riga, Latvia; Baltic HIV Association, Riga, Riga, Latvia; Riga East University Hospital, Rīga, Riga, Latvia; Riga East University Hospital, Rīga, Riga, Latvia

## Abstract

**Background:**

According to previous epidemiological studies there is 2.4% prevalence of anti-HCV in Latvian population. Hepatitis C can lead to liver cirrhosis and HCC. There is available an effective HCV treatment with 100% reimbursement. WHO has settled a goal – to eliminate HCV as a public health threat by 2030. The biggest obstacle to achieve this goal is to find an infected persons.

The aim of this study was to screen and analyze the data of patients from Psychiatry hospitals (PH) in Latvia. Patients were screened for hepatitis C infection (anti-HCV) and analyze the possible risk factors.

**Methods:**

795 patients of all PH in Latvia were tested, including 57.1% males and 42.9% females. The mean age was 49 years (range 18 - 93). Data were obtained by performing survey and rapid blood antibody tests. The questionnaire included demographic information and 10 questions on infection risk factors. Study was done from May till October 2021 by HIV Prevention Point workers in PHs of 7 PH in Latvia – Riga, Daugavpils, Liepaja, Strenci, Gintermuiza, Akniste and Vecpiebalga. Data were analyzed using MS Excel and IBM SPSS.

**Results:**

24.8% of patients noted they have been sexually active in the last year, but only 9.9% admitted usage of condoms. 3.8% noted they are or have been used IV drugs. 11.2% of patients have a history of incarceration.

In total 6.8% of performed tests were positive for anti-HCV. Incarcerated persons were positive in 16% of cases, non-incarcerated – in 6%. IV drug users (IVDU) were positive in 49%, non-users – in 4.9%, all differences are statistically significant.

**Conclusion:**

Patients in PH are at higher risk of HCV infection in comparison to population in Latvia. Besides already well known risk groups – IVDU and incarcerated people, we found 2.8 times higher anti-HCV prevalence in psychiatric hospital’s patients, with markedly higher prevalence in those previously incarcerated or IVDU. This study provides valuable data to better identify groups of individuals with higher risk of HCV infection. Latvia. PNC patients should further be tested, especially individuals who have been incarcerated, use or have used IV drugs.

**Disclosures:**

**All Authors**: No reported disclosures.

